# Impact of artificial intelligence on electronic health record-related burnouts among healthcare professionals: systematic review

**DOI:** 10.3389/fpubh.2025.1628831

**Published:** 2025-07-03

**Authors:** Berna Sarraf, Ali Ghasempour

**Affiliations:** ^1^Department of Health Technology, School of Information Technology, Tallinn University of Technology, Tallinn, Estonia; ^2^School of Information Technology, IT College, Tallinn University of Technology, Tallinn, Estonia

**Keywords:** artificial intelligence, electronic health records, burnout, healthcare professionals, workload

## Abstract

**Introduction:**

The implementation of electronic health records (EHRs) has revolutionized modern clinical practice, increasing efficiency, accessibility, and quality of care. Nevertheless, EHR-related workload has been considered as a significant contributor to healthcare professionals’ burnout, a syndrome associated with emotional exhaustion, depersonalization, and reduced personal accomplishment. As modern health system explores technological solutions, artificial intelligence (AI) has gained attention for its potential to facilitate documentation processes and alleviate cognitive burden. This systematic review aims to explore and understand the impact of artificial intelligence on burnout associated with electronic health records among healthcare professionals.

**Methods:**

A systematic literature review was conducted following the PRISMA 2020 guidelines. Relevant studies published between 2019 and 2025 were retrieved from three electronic databases: PubMed, Scopus, and Web of Science. The search strategy included three main domains: artificial intelligence, electronic health records, and healthcare professional burnout. Eligible included studies are peer-reviewed original research articles that evaluated the impact of AI-based technologies on burnout among healthcare professionals. The screening and selection processes were carried out by following the PRISMA framework. Methodological quality assessment of the included studies was performed using the Joanna Briggs Institute Critical Appraisal Tools.

**Results:**

Of the 287 records initially identified, eight studies met the inclusion criteria. The majority of identified studies were conducted in the United States and Canada. The identified interventions were categorized into four domains: ambient artificial intelligence scribes, clinical decision support systems, large language models, and natural language processing tools. Most studies focused on mitigating documentation or inbox-related burdens and reported positive outcomes, including decreased documentation time, enhanced workflow efficiency, and reduced symptoms of burnout among healthcare professionals. Nonetheless, several methodological limitations were observed, including the absence of control groups, small sample sizes, and short follow-up periods, which constrain the generalizability of the findings.

**Discussion:**

The integration of artificial intelligence into electronic health record systems may have potential to alleviate documentation burden and inbox management burden. Although preliminary findings are promising, further methodologically robust research is necessary to evaluate long-term outcomes, assess usability across diverse clinical contexts, and ensure the safe and effective implementation of AI technologies in routine healthcare practice.

**Systematic review registration:**

https://osf.io/pevfj.

## 1 Introduction

In our contemporary world, the healthcare environment is undergoing continuous transformation, which results in both incremental improvements and disruptive innovations (1). These changes are reshaping clinical workflows, altering provider responsibilities, and redefining patient and caregiver experiences (1, 2). One of the most significant technological advancements driving this transformation is the widespread adoption of Electronic Health Records (EHRs). An EHR is defined as a system for collecting and storing patient health data in a digital environment (3). This data typically includes demographics, diagnoses, medications, vital signs, laboratory results, medical history, immunization records, and radiology reports (4, 5). The implementation of EHRs has shown to improve healthcare service quality by reducing medication errors, promoting adherence to clinical guidelines, and enhancing operational efficiency (6, 7).

Despite the many benefits promised by EHR systems, their implementation has also introduced substantial challenges that necessitate critical evaluation. One of the main issue among these is the increased workload associated with EHR use, as healthcare professionals are required to spend significant time documenting, reviewing, and summarizing patient data (8–11). This administrative burden is not only time-consuming but has been identified as a key contributor to healthcare professional burnout (12), a psychological syndrome that develops in response to prolonged occupational stress (13). Burnout has been linked to adverse outcomes for physician well-being, increased workforce attrition, and compromised patient care, including reduced care quality and safety (2, 12, 14, 15). Burnout can lead to serious consequences for healthcare professionals, particularly physicians, among whom suicide rates are twice as high as in the general population (16, 17).

High workload is a well-recognized occupational stressor directly affecting care quality and patient outcomes. Prior studies have shown that administrative tasks, particularly those related to EHR use, substantially increase staff workload and time pressure (18). For example, in ambulatory care settings, physicians spend approximately 49% of their time on EHRs and desk work, compared to just 33% on direct clinical interactions with patients and staff (1, 19). This imbalance highlights the extent to which digital documentation demands interfere with patient care. Furthermore, recent surveys show that up to 70% of clinicians experience stress linked to the use of health information technology, with EHR-related stress emerging as an independent predictor of burnout (20). In this regard, AI-driven solutions are increasingly being explored as a means to alleviate the EHR-associated burnout.

Over the past decade, Artificial Intelligence (AI) has evolved from a theoretical concept into a practical and increasingly integrated component of modern healthcare systems (21). The rapid and exponential growth of AI applications introduces an important opportunity to address long-standing challenges in clinical practice, including clinician burnout and increased workload (21). AI encompasses a range of advanced computational methods such as Natural Language Processing (NLP), deep learning, intelligent robotics, and context-aware computing (22). Unlike traditional analytics, which operate on predefined rules, AI systems possess the ability to learn from historical data, adapt over time, and simulate human cognitive functions (22, 23).

In healthcare, AI has already demonstrated utility in various domains, including automating administrative workflows, enhancing diagnostic accuracy, supporting clinical decision-making, designing personalized treatment plans, and guiding robotic-assisted surgeries (23–26). When integrated with EHRs, AI technologies can facilitate data entry, retrieve relevant clinical information, and even transcribe patient-clinician interactions in real time (9, 27). These capabilities hold substantial promise for reducing the time health care professionals spend on manual documentation, thereby improving workflow efficiency and potentially mitigating the risk of burnout. While AI’s clinical applications continue to expand, its role in alleviating EHR-related burnout remains an underexplored yet promising area of investigation that necessitate further study (28).

### 1.1 Research aim

The aim of this study is to systematically review existing literature to explore and understand the impact of artificial intelligence integration on burnout associated with electronic health records among healthcare professionals.

### 1.2 Research question

In what ways can artificial intelligence integration into electronic health record systems reduce burnout among healthcare professionals?

## 2 Study method

### 2.1 Study design

The design for this study is a systematic review, which aims at critically appraising and synthesizing the existing literature on the integration of AI in EHR systems to reduce burnout among healthcare professionals. The review will identify, evaluate, and summarize published studies exploring the effectiveness, challenges, and potential strategies involved in leveraging AI to allaviate EHR-related burnout. Additionally, PRISMA guidelines (Preferred Reporting Items for Systematic Reviews and Meta-Analyses) were followed for the systematic review (29).

### 2.2 Search strategy and databases

To capture the latest developments in the emerging field of AI technologies in healthcare, a literature search was conducted across multiple interdisciplinary databases, including PubMed, Scopus, and Web of Science. Although IEEE Xplore was initially considered due to its strong focus on technical and engineering research, it was ultimately excluded from the final selection. This decision was based on its limited relevance to the central themes of our study, which emphasized health workforce outcomes, usability, and the mental health implications of AI implementation in clinical environments. Since IEEE Xplore predominantly features content on system design and technical innovation, it was less suited to our investigation into the practical and occupational impact of AI-driven EHR systems on healthcare professionals. In contrast, PubMed, Scopus, and Web of Science offered a broader and more clinically oriented evidence base, better aligned with the goals of the review.

A narrative synthesis approach was employed to summarize findings across studies, with a particular focus on the role of AI in reducing burnout associated with EHR use among healthcare professionals. The search was performed between February and April 2025. Given that initial keyword combinations in PubMed indicated that relevant studies were available from 2019 onward, the search was limited to publications from 2019 to 2025. Three main concepts were identified for the search strategy: Artificial Intelligence (AI), Electronic Health Records (EHR), and Burnout Among Healthcare Professionals. To ensure comprehensiveness, the search terms were broadened by searching their synonyms. For the first concept, the synonyms identified included “Machine Learning” OR “Deep Learning” OR “Natural Language Processing” OR “Clinical Decision Support System.” The second concept used the terms “Health Information System” OR “Electronic Medical Record” OR “EHR” OR “Clinical Documentation System” OR “Health IT” OR “Digital Health Record.” The synonyms of the third concept included “Burnout” OR “Job Burnout” OR “Occupational Stress” OR “Professional Burnout” OR “Mental Fatigue” OR “Clinician Burnout” OR “Physician Burnout” OR “Nurse Burnout” OR “Occupational Stress.” The complete search strategy, including Boolean operators, database-specific filters, and search results, is outlined in Table 1.

**Table 1:** Overview of literature search strategy across databases.

Database	Search string/Boolean logic	Date of last research	Filters applied	Results retrieved	Included in final review
PubMed	(“Artificial Intelligence”[MeSH] OR “AI”[MeSH] OR “Machine Learning”[MeSH] OR “Deep Learning” OR “Natural Language Processing” OR “Predictive Analytics”[MeSH] OR “Clinical Decision Support System” OR “Automation in Healthcare”) AND (“Electronic Health Records”[MeSH] OR “EHR”[MeSH] OR “Health Information System” OR “Electronic Medical Record” OR “EHR”) AND (“Burnout, Professional”[MeSH] OR “Occupational Stress” OR “Mental Fatigue” OR “Clinician Burnout” OR “Physician Burnout”)	2025-04-10	English, 2019–2025, Peer-reviewed	18	Yes
Scopus	TITLE-ABS-KEY ((“Artificial Intelligence” OR “Machine Learning” OR “Deep Learning” OR “Natural Language Processing” OR “Predictive Analytics”) AND (“Electronic Health Record” OR “EHR” OR “Health Information System”) AND (“Burnout” OR “Job Burnout” OR “Workload” OR “Mental Fatigue” OR “Clinician Burnout” OR “Physician Burnout” OR “Occupational Stress”))	2025-04-10	English, 2019–2025, Peer-reviewed	236	Yes
Web of Science	(“Artificial Intelligence” OR “Machine Learning” OR “Deep Learning” OR “Natural Language Processing” OR “Predictive Analytics”) AND (“Electronic Health Record” OR “EHR” OR “Clinical Decision Support System” OR “Health Information System”) AND (“Burnout” OR “Workload” OR “Healthcare Professional Stress” OR “Mental Fatigue”)	2025-04-10	English, 2019–2025, Peer-reviewed	33	Yes
IEEE Xplore	(“Artificial Intelligence” OR “Machine Learning” OR “Deep Learning” OR “Natural Language Processing” OR “Predictive Analytics”) AND (“Electronic Health Record” OR “EHR” OR “Clinical Decision Support System” OR “Health Information System”) AND (“Burnout” OR “Workload” OR “Healthcare Professional Stress” OR “Mental Fatigue”)	2025-02-16	English, 2019–2025, Conference & Journal Articles (but most not peer-reviewed)	14	No

### 2.3 Quality assessment

The methodological quality of the included studies was assessed using the Joanna Briggs Institute (JBI) Critical Appraisal Tools appropriate to each study design (30). The aim of this appraisal is to judge their methodological rigor and assess how well they have minimized potential bias in their design, execution, and analysis (31). Two reviewers independently appraised each study using the relevant JBI tool to evaluate potential sources of bias, methodological rigor, and overall validity. Any disagreements were resolved through discussion. The quality assessment helped to inform the interpretation of the review findings, but no studies were excluded solely based on quality scores. Consequently, two different JBI critical appraisal tools were employed based on study design. The Checklist for Quasi-Experimental Studies was used for six studies that applied pre-post or observational interventions without randomization (31). These appraisals are summarized in Table 2. For the two studies that followed a cross-sectional analytical design, the Checklist for Analytical Cross-Sectional Studies was used to evaluate risk of bias and methodological rigor (32), as shown in Table 3.

**Table 2:** JBI critical appraisal for quasi-experimental studies.

Checklist for quasi-experimental studies	Q1	Q2	Q3	Q4	Q5	Q6	Q7	Q8	Q9	Score
Shah et al. (33)	Yes	No	Yes	Yes	Yes	Yes	Yes	Unclear	Yes	7/9 (78%)
Laing and Mercer (37)	Yes	No	Yes	Yes	Yes	Yes	Yes	Yes	Yes	8/9 (89%)
Yang et al. (40)	Yes	No	Yes	Unclear	Yes	Yes	Yes	Yes	Yes	7/9 (78%)
Garcia et al. (39)	Yes	No	Yes	Yes	Yes	Yes	Yes	Yes	Yes	8/9 (89%)
Barak-Corren et al. (38)	Yes	No	Yes	Yes	Yes	Yes	Yes	Yes	Yes	8/9 (89%)
Albrecht et al. (35)	Yes	No	Yes	Yes	Yes	Yes	No	Yes	Yes	7/9 (78%)

**Table 3:** JBI critical appraisal for analytical cross-sectional studies.

Checklist for analytical cross-sectional studies	Q1	Q2	Q3	Q4	Q5	Q6	Q7	Q8	Score
Owens et al. (34)	yes	yes	yes	yes	yes	yes	yes	yes	8/8 (100%)
Joseph Moryousef et al. (36)	no	yes	yes	yes	no	no	yes	yes	5/8 (63%)

### 2.4 Data screening and analysis

Screening was conducted in two phases: an initial screening of titles and abstracts, followed by a full-text review. Both phases were independently performed by two reviewers to ensure objectivity and rigor. Any discrepancies between reviewers were discussed and resolved through weekly team meetings. No automation tools were employed, and no authors were contacted for additional information. During the identification phase, the PRISMA flow diagram was utilized to systematically map the number of records identified, screened, included, and excluded (29). To ensure comprehensive reporting and methodological rigor, the PRISMA 2020 Checklist was used throughout the entire review process, covering the introduction, methods, results, and discussion sections (29).

**Figure 1:**
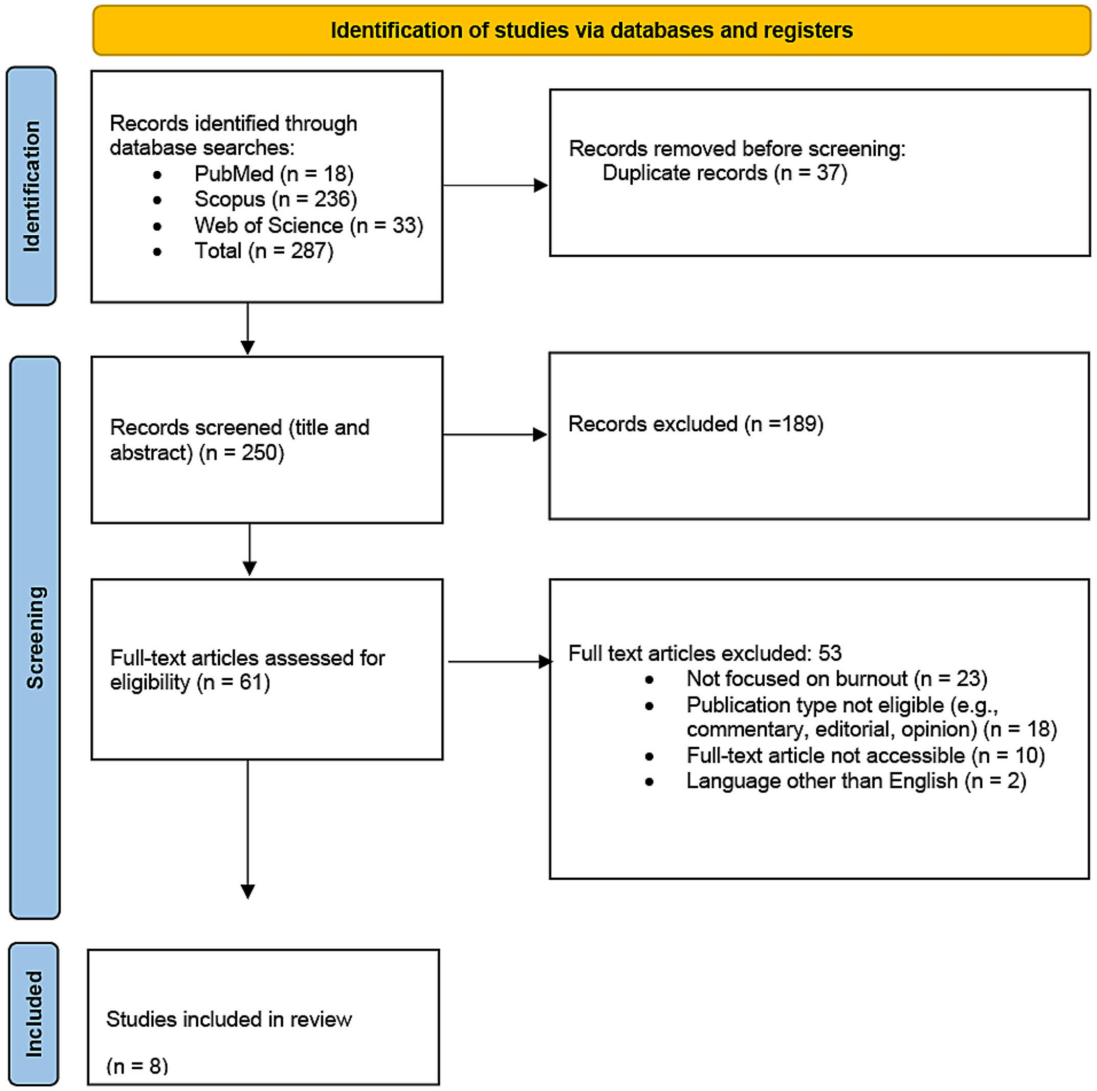
PRISMA 2020 flow diagram for new systematic reviews which included searches of databases and registers only.

A total of 287 articles were identified through multiple database searches, and of these, 37 (12.89%) duplicates were removed. This resulted in 250 (87.1%) articles. The first screening was conducted based on the titles and abstracts of the articles, with inclusion and exclusion criteria applied by the authors. This review includes original, peer-reviewed research studies (quantitative, qualitative, or mixed methods) published in English between 2019 and 2025. Studies must focus on healthcare professionals, including physicians, nurses, allied health workers, and other clinicians. The inclusion and exclusion criteria are detailed in Table 4. These 250 articles were randomly divided into two batches, which were assigned to the researchers seperately. As a result of the first screening, 75.6% (189/250) of articles were excluded, resulting in 61 (24.4%) articles. Following an approach to the first screening, the second screening involved a full-text review by the authors to ensure that the articles met all inclusion criteria, with particular focus on study design and focus on AI and burnout. The 61 articles were again randomly divided into two batches and assigned to each researcher. The assessment of each of the 61 articles was then verified by the research team, resulting in a final set of 8 (13.11%) relevant articles. During the second screening phase, weekly meetings were held with the research team in which any uncertainties were raised and discussed until consensus was reached. Overall, the final screening resulted in the inclusion of 8 articles. The full process is mapped by the PRISMA 2020 framework, as illustrated in Figure 1.

**Table 4:** Inclusion and exclusion criteria.

Inclusion criteria	Exclusion criteria
Language: English	Studies published in languages other than English
Publication date: 2019–2025	Studies published before 2019
Study type: Peer-reviewed articles Original research (quantitative, qualitative, or mixed methods)	Study type: Systematic reviews, opinion/editorial pieces, non–peer-reviewed articles, abstract-only papers, conference materials, books, and dissertations
Population: Healthcare professionals, including physicians, nurses, allied health workers, other clinicians using EHR systems	Studies not involving healthcare professionals (e.g., IT specialists, administrators, or patients)
Outcomes: Studies assessing outcomes related to documentation burden, efficiency, usability, or burnout-related factors (e.g., time saved, workflow improvement, satisfaction, stress).	Outcomes: Studies that do not evaluate provider-facing outcomes or focus only on system-level metrics without human-level burden, usability, or wellness endpoints.

Extracted data included key study characteristics such as author and publication year, the specific problem addressed, the type of AI intervention used, and contextual details including study setting, clinical specialty, and study design. Additionally, outcomes such as key findings, reported challenges, and suggested directions for future research were documented. This structured extraction approach allowed for consistent comparison across studies. Given the exploratory nature of this review, which aim to descriptively synthesize findings across diverse study types, no quantitative effect estimates (e.g., odds ratios or risk ratios) were calculated, and no meta-analysis was performed. A formal risk of bias assessment was not conducted due to the methodological heterogeneity and descriptive intent of the included studies. Additionally, reporting bias and certainty of evidence (e.g., using the GRADE approach) were not formally assessed, as they were not applicable to the objectives and design of this exploratory synthesis. A completed PRISMA 2020 Checklist is included as a Supplementary document.

## 3 Results

The articles included in this systematic review were published between 2019 and 2025. Most originated from the United States (*n* = 6), with two additional studies conducted in Canada. The majority targeted healthcare professionals, primarily physicians, with sample sizes ranging from 10 to 162 participants. Across the eight studies, four categories of AI-based interventions were identified: Ambient AI scribes (*n* = 4), Clinical Decision Support Systems (CDSS; *n* = 1), Large Language Models (LLMs; *n* = 2), and Natural Language Processing (NLP) summarization tools (*n* = 1). These interventions were evaluated using a range of study designs, including prospective quality improvement studies, observational studies, mixed-methods evaluations, pre-post survey designs, comparative simulation-based analyses, and analytical cross-sectional studies.

**Figure 2:**
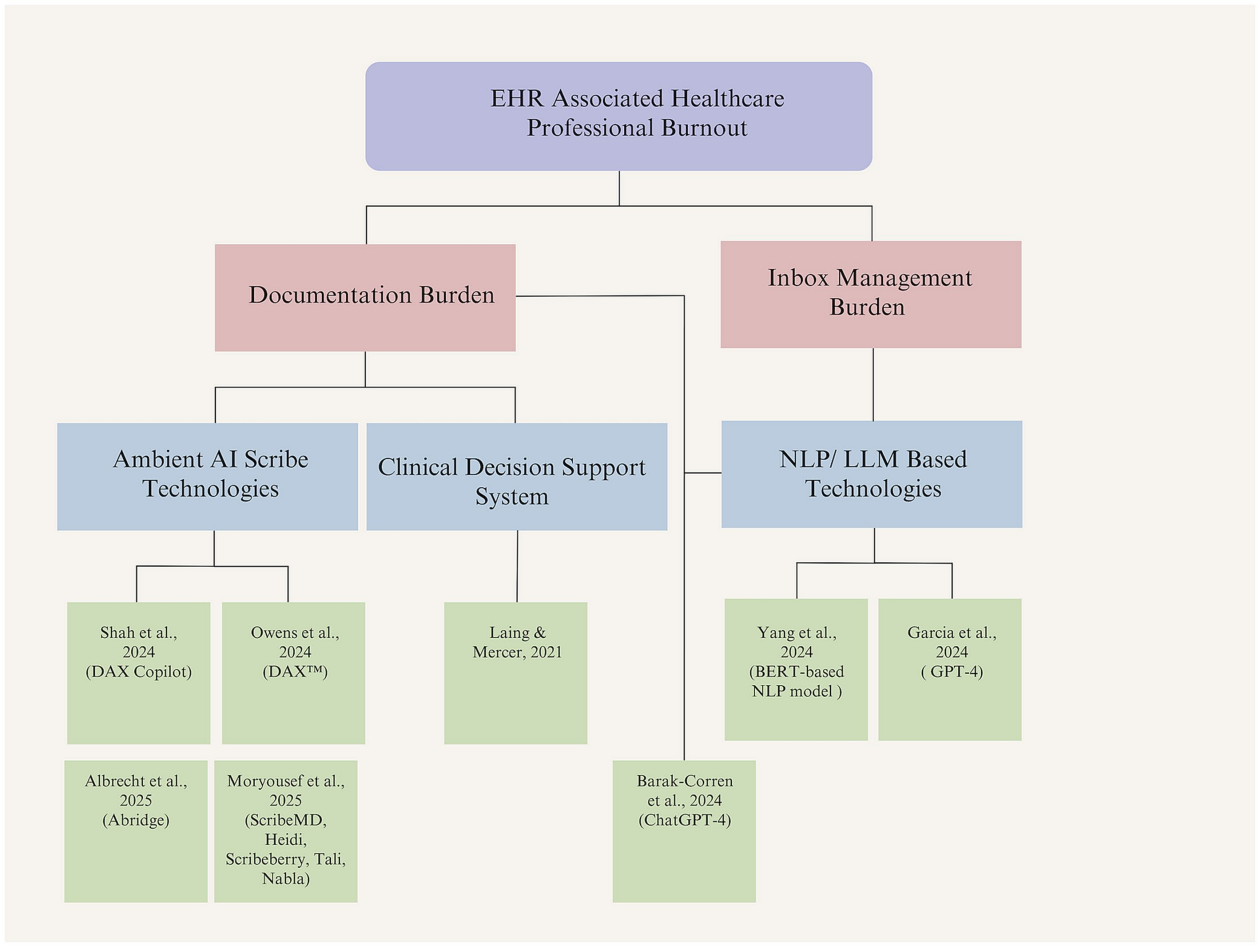
Classification of included studies by AI intervention and addressed problem (created by authors).

To synthesize the findings thematically, the studies were categorized not only by technology type but also by the specific EHR-related challenges they addressed. Two main subproblems emerged as contributors to healthcare professional burnout: documentation burden and inbox management burden. Documentation burden was defined by excessive time spent on clinical notes, inefficient workflow integration, and reduced time for patient interaction. Six studies addressed this issue, including those focused on ambient AI scribes, a CDSS tool, and one LLM-based intervention. The second subproblem, inbox management burden, stemmed from high volumes of patient portal messages, poor triage systems, and a lack of time-saving tools. Two studies in the review used NLP and LLM technologies to address this challenge. Figure 2 presents a visual classification of the included studies according to these problem domains and AI intervention types.

### 3.1 Methodological quality appraisal of included studies

The methodological quality of the included studies was assessed using JBI Critical Appraisal Checklists appropriate to each study design. Six studies were evaluated using the JBI Checklist for Quasi-Experimental Studies, while two other studies were assessed using the JBI Checklist for Analytical Cross-Sectional Studies.

Among the quasi-experimental studies, methodological quality was generally rated as moderate to high. Most studies demonstrated a clearly established temporal relationship between the intervention and observed outcomes, employed appropriate statistical analyses, and consistently measured outcomes. However, a notable limitation across all six quasi-experimental studies was the absence of a control group (Q2). Additionally, several studies did not adequately report on follow-up completeness (Q8) and lacked the use of validated outcome measures (Q7), which may affect the reliability of findings.

Of the two cross-sectional studies, one met all the appraisal criteria and was deemed high in methodological quality. The second study was rated as moderate due to insufficient or unclear reporting on inclusion criteria and the identification and control of confounding variables. Overall, while the studies generally demonstrated methodological soundness, limitations related to design rigor and reporting transparency should be taken into consideration when interpreting the findings. Tables 2, 3 present a detailed summary of the JBI critical appraisal results for all included studies.

### 3.2 Synthesis of results by intervention type

#### 3.2.1 Ambient AI scribe technologies

To address the burnout stemming from EHR documentation burden, four studies proposed the use of ambient AI technologies. Shah et al. conducted a prospective quality improvement study at Stanford Health Care to assess the DAX Copilot, an ambient AI scribe integrated with the Epic EHR system (33). Ambient AI refers to AI systems that work in the background to capture conversations and automatically generate clinical documentation without requiring active input (33). The tool is designed to alleviate documentation workload and its contribution to physician burnout. It captures clinician-patient interactions and automatically generates clinical notes. Similarly, Owens et al. conducted an observational study at the University of Michigan Health-West to evaluate the impact of DAX™, an ambient voice-enabled AI documentation tool, on primary care provider burnout and documentation burden (34).

The former study measured usability, perceived utility, and the impact on documentation workload and burnout. The results demonstrated a significant reduction in task load and burnout, alongside increased usability and efficiency. While these findings are promising, the authors emphasized the need for further research involving diverse populations and objective metrics to support broader adoption and long-term integration. The latter study, using survey data from 83 providers and electronic medical record analytics, found that high DAX™ usage (>60% of encounters) was significantly associated with reduced burnout on the disengagement subscale of the Oldenburg Burnout Inventory, although not on the exhaustion or total scores. Additionally, DAX™ usage led to a 28.8% reduction in documentation time per encounter and decreased after-hours charting.

Another study addressing the similar issue was conducted by Albrecht et al., who carried out a quality improvement study at the University of Kansas Medical Center to evaluate the impact of Abridge, an ambient AI documentation platform, on clinician workflow, documentation burden, and well-being (35). Using pre-and post-implementation surveys among 181 clinicians across 30 specialties, the study found that Abridge significantly improved perceptions of workflow ease (OR = 6.91) and the likelihood of note completion before the next visit (OR = 4.95). Most clinicians reported reduced after-hours documentation, a lower risk of burnout, and increased job satisfaction, regardless of specialty or duration of use.

Finally, Moryousef et al. conducted a comparative evaluation of five freely accessible AI scribes to assess their efficacy and clinical utility in urologic documentation (36). The study used simulated encounters for common urology referrals and compared AI-generated notes against standardized reference notes, with quality assessed by 20 Canadian urologists. Among the five freely accessible AI scribes, Nabla and Tali emerged as the most favorable tools. Nevertheless, all scribes exhibited errors, which raised concerns regarding accuracy and patient safety. While 75% of respondents identified documentation as a major source of burnout, 90% expressed openness to adopting AI scribes. The study highlights the potential of these tools to alleviate administrative burden and improve clinician-patient interactions, while also emphasizing that such tools should support rather than replace clinician documentation.

Overall, the sudies reveal that ambient AI scribe technologies can meaningfully reduce administrative workload and improve aspects of provider well-being. However, further longitudinal and controlled studies are recommended to assess broader impacts and generalizability. Also, the studies indicate the need for improving accuracy, ensuring medico-legal compliance, and evaluating performance across diverse and complex clinical settings. Table 5 summarizes the articles that focus on ambient AI scribe technologies aimed at addressing the EHR documentation burden.

**Table 5:** Summary of ambient AI scribe studies.

Study	Problem addressed	AI tool(s) used	Setting	Specialty	Study design	Key findings	Challenges	Future research
Shah et al. (33)	EHR documentation burden	DAX Copilot	Stanford Health Care	Primary Care	Prospective Quality Improvement Study	Large statistically significant reductions in task load (−24.42, *p* < 0.001) and burnout (−1.94, *p* < 0.001); moderate statistically significant improved usability (+10.9, *p* < 0.001); positive utility outcomes	Recruitment; integration with existing workflows; rapid technological progression; diverseness of fit	Broader studies with qualitative interviews and mixed-method approaches; incorporating the patient perspectives
Owens et al. (34)	EHR documentation burden	DAX™	University of Michigan Health-West	Primary Care	Observational Study	High use of DAX™ (>60% of encounters) among 28% (23/83) of the respondents; lower burnout measured by OLBI disengagement sub-score (mean difference [MD] − 2.1; 95% confidence interval [CI]: −3.8 to −0.4); no significant differences in the OLBI exhaustion sub-score (MD − 1.0; 95% CI: −2.9 to 1.0) or the total OLBI score (MD − 3.0; 95% CI: −6.4 to 0.3); reduction in documentation time by 28.8% (1.8 min; 95% CI: 1.4 to 2.2)	Short follow-up; self-selection bias; unidentified confounders; generalizability to other primary care cohorts (limitation); predicting the provider characteristics for high adoption	Long-term, controlled studies with objective data and broad group of primary care providers
Albrecht et al. (35)	EHR documentation burden	Abridge	University of Kansas Medical Center	Multi-specialty	Pre-post Survey Study	Significant improvement in clinician documentation workflow (OR = 6.91, 95% CI: 3.90–12.56, *p* < 0.001) and completing notes (OR = 4.95, 95% CI: 2.87–8.69, *p* < 0.001); reduced after-hours work; higher job satisfaction	Survey differences; limited objective metrics; early adopter bias	Objective measurements of AI documentation technologies (e.g., time spent on documentation, note quality, time spent outside of work, financial effects); longer-term impact; comparison of emerging AI ambient tools and traditional methods; clinician readiness; evaluating the impact on different stakeholders
Moryousef et al. (36)	EHR documentation burden	ScribeMD, Heidi, Scribeberry, Tali, Nabla	Canadian Academic Urology Programs	Urology	Comparative Evaluation Study	Found clinical documentation as a significant source of burnout (75% of the respondents); 90% reported openness to using AI scribes; Nabla performed the best (with a favorable score of 68% and lowest critical error score of 28%); all tools demonstrated various minor errors and critical errors	Documentation accuracy; hallucination errors; automation bias; medico-legal and privacy concerns; multicultural and multilinguistic considerations (e.g., different accents, dialects, and languages)	Improvements of AI models for hallucination errors, accuracy and safety issues; Evaluation in complex/multilingual settings; inclusion of paid tools; longitudinal studies

#### 3.2.2 Clinical decision support system

One article examined the application of CDSS as a strategy to mitigate EHR-related documentation burden. Laing and Mercer conducted a pre-post simulated study to evaluate the impact of a point-of-care CDSS integrated within an EMR on preventive care documentation efficiency (37). Seventeen clinicians from a Canadian family health team assessed artificial patient charts with and without the CDSS, measuring time, accuracy, and perceived usability. The CDSS reduced chart review time by an average of 195.6 s without compromising decision accuracy. Participants rated the tool highly in terms of usability and usefulness, citing improvements in both efficiency and organizational workflow. Although limitations regarding generalizability and study design were noted, the findings indicate that EMR-integrated CDSSs hold potential for enhancing preventive care processes and alleviating documentation burden in clinical practice. A summary of this study is presented in Table 6.

**Table 6:** Summary of CDSS study.

Study	Problem addressed	AI tool(s) used	Setting	Specialty	Study design	Key findings	Challenges	Future research
Laing and Mercer (37)	EHR documentation burden	Point-of-care CDSS integrated in PS Suite EMR	Bruyère Family Health Team (Canada)	Primary Care	Pre-post simulated evaluation	Reduced chart review time by 195.6 s (249.5 s vs. 445.2 s; *P* < 0.001); potential to save 82.6 h per year; no loss in decision accuracy (78.4% vs. 80.9%, *p* > 0.05); high usability scores	Interface concerns (e.g., confusing date formats); the inherent trust in the CDSS; data accuracy concerns; layout usability	Real-world testing across settings and EMRs; improved design based on user feedback or in-depth workflow analysis; longitudinal impact

#### 3.2.3 Large language models and natural language processing

Three studies were categorized under technologies adopting LLMs and NLP for clinical applications. One study addressed EHR-related documentation burden through the use of LLMs, while the remaining two focused on alleviating the burden associated with inbox message management by leveraging LLM and NLP-based tools.

Barak-Corren et al. conducted a mixed-methods study to evaluate the feasibility of using ChatGPT-4 for generating clinical documentation in pediatric emergency medicine (38). Ten emergency physicians reviewed AI-generated supervisory notes, I-PASS handoffs, and patient-facing letters across simulated cases. In complex scenarios, ChatGPT reduced documentation time by up to 43% and effort by 33%, with summaries rated highly for accuracy, readability, and efficiency. Most participants supported adoption for complex cases but raised concerns about liability, clinical voice, and integration into workflows. The study highlights the promise of generative AI in easing documentation burden, necessitating further validation in real-world settings.

Garcia et al. conducted a 5-week quality improvement study at Stanford Health Care to evaluate the use of GPT-4–generated draft replies to patient messages within the EHR (39). Among 162 clinicians in primary care and gastroenterology, the AI tool achieved a 20% mean utilization rate and was associated with significant reductions in perceived task load and work exhaustion. Although no measurable reductions in reply time were observed, clinicians reported high usability and variable satisfaction depending on role. Challenges pointed out include limited personalization, basic patient context, and uneven adoption. The study supports the potential of a LLM–driven tools to reduce clinician burden. It also highlights the need for further evaluation through controlled, multisite research.

Finally, Yang et al. developed and implemented a BERT-based artificial intelligence model to prioritize high-acuity patient portal messages within the Epic EHR at NYU Langone Health (40). Trained on over 40,000 messages, the model flagged urgent messages to help registered nurses’ triage more efficiently. In a pre-post observational study analyzing 396,466 messages, the system reduced median read time for flagged high-acuity messages by 9 min during business hours and 21 min during non-business hours. The model achieved strong performance (AUC 0.97, precision 67%, sensitivity 63%) and was well-integrated into clinical workflows. Despite limitations in evaluation design and measurement of clinical outcomes, findings support AI-enabled message prioritization as a promising tool to enhance care responsiveness and reduce triage burden. The study recommends future research with rigorous designs and broader settings. Table 7 provides a summary of the studies that employed LLM and NLP based technologies to address EHR-related documentation and inbox management burdens.

**Table 7:** Summary of studies using LLM and NLP based technologies.

Study	Problem addressed	AI tool(s) used	Setting	Specialty	Study design	Key findings	Challenges	Future research
Yang et al. (40)	Inbox management burden	BERT-based NLP model for message acuity detection	NYU Langone Health	Care Coordination/Primary Care	Pre-post quality improvement study	Reduced read time by up to 21 min for urgent messages; strong model performance (C-statistic = 97%, average-precision = 72%)	Workflow/staffing confounders; limited resolution time measurement; randomization issues	Implementation of the tool in different settings; conducting more sophisticated study designs; improving scoring visibility; measuring patient outcomes
Garcia et al. (39)	Inbox management burden	GPT-4 for auto-generating patient message replies	Stanford Health Care	Primary Care and Gastroenterology	5-week prospective quality improvement study	20% draft utilization; reduced task load and exhaustion; positive usability across most roles; no change in reply action time	Inconsistent adoption; limited personalization; lack of model fine-tuning	Multi-site trials; model training on domain data; patient outcome and perception studies
Barak-Corren et al. (38)	EHR documentation burden	ChatGPT-4 for clinical summaries, I-PASS, patient letters	Pediatric Emergency Department (academic)	Pediatric Emergency Medicine	Mixed-methods proof-of-concept study	40% reduction in time and 33% decrease in efforts for complex cases; high ratings for completeness and readability	Differential diagnosis; hallucination; privacy and compliancy issues; legal liability; loss of clinical voice; limited real-time integration; potential increase in the volume and length of documentation	Blinded comparisons; clinical outcome metrics; workflow-embedded secure tools; further implementation studies to address AI-related concerns

## 4 Discussion

This systematic review identified two primary subdomains contributing to EHR-related burnout among healthcare professionals: documentation burden and inbox management burden. These subdomains represent important targets for AI-based interventions, which this study explores as potential solutions to alleviate burnout. Prior studies that focused on quantifying and categorizing the sources of EHR-related workload further support these findings. For instance, Arndt et al. conducted a time-motion study using EHR event log data and observed that primary care physicians spent nearly 6 h per workday interacting with the EHR, with documentation and inbox tasks accounting for 44.2 and 23.7% of EHR time, respectively (8). Their taxonomy outlined 15 EHR-related activities and emphasized documentation, chart review, and inbox management as the most time-consuming (8). Similarly, a study reviewed the literature on physician burnout and classified EHR-related contributors into several categories, including documentation and clerical burdens, cognitive overload, electronic messaging volume, and complex usability issues (18).

Research shows that EHR documentation burden is a major contributor to healthcare professional frustration and burnout (12). Many healthcare professionals are unable to complete documentation tasks during clinic hours. As a result, they use personal time to finish their work (9, 41). This increased workload contributes to reduced job satisfaction (14), less time for patient interaction (2), and may negatively impact the quality of care provided (12). To address this issue, this review identified four AI interventions, including ambient AI scribe technologies, CDSS, and LLM and NPL based technologies. Among the AI-based interventions identified in this review, ambient AI scribe technologies emerged as a promising approach to alleviating EHR documentation burden. These tools, such as DAX Copilot, Abridge, ScribeMD, and others, advantage real-time speech recognition and NLP to transcribe clinician-patient conversations simply, automating the generation of clinical notes. Across multiple studies, these systems demonstrated evident benefits including reduced after-hours documentation, improved workflow efficiency, enhanced clinician satisfaction, and even modest reductions in self-reported burnout. For example, Shah et al. and Owens et al. found improvements in usability and task load (33, 34), while Albrecht et al. reported increased satisfaction and reduced after-hours charting (35).

While ambient AI scribe technologies offer promising solutions to alleviate documentation burden, recent findings caution against early adoption of AI-generated notes in clinical practice without rigorous evaluation. A study employing standardized simulated patient-provider interactions found that clinical notes generated by ChatGPT-4 failed to meet acceptable quality standards for clinical use (2). Notably, the study revealed substantial variability in error types, including both errors of omission and commission, which were not confined to specific sections of the SOAP note (2). Moreover, inconsistencies in error patterns across identical case replicates suggest that the model introduces an element of unpredictability, which makes it difficult for healthcare professionals to anticipate or correct errors reliably (2). This unpredictability presents a risk to clinical oversight and safety. It demonstrates the need for rigorous quality assurance before integrating AI technologies into routine documentation workflows (2). Integration into existing clinical workflows also presents logistical and technical difficulties, and the rapid pace of technological evolution may overtake institutions’ ability to adapt effectively (2, 9, 27). Moreover, most current studies relied on short-term, subjective measures (e.g., surveys), limiting their generalizability and robustness. The lack of patient perspectives and validated outcome metrics further constrains the understanding of their true impact.

While the majority of studies in this review focus on physicians, it is critical to recognize that nurses are also significantly impacted by EHR-related documentation burden. Nurses represent one of the largest group of EHR end-users in healthcare and routinely document 600–800 data points per 12-h shift, equating to roughly one data point per minute (14, 42, 43). Despite this, their experiences are underrepresented in burnout research and AI intervention design. A study by Gesner et al. provides strong evidence that documentation burden in nursing is positively correlated with emotional exhaustion and depersonalization, two key dimensions of clinician burnout syndrome (14). Moreover, the study identifies poor EHR usability as a significant contributor to documentation burden and calls for improved system design tailored to nursing workflows (14). The authors emphasize that while AI-based interventions such as clinical decision support and voice recognition tools hold promise, nurse-centered design and evaluation are essential to ensure these technologies effectively reduce burden without introducing new risks or inefficiencies (14). These findings highlights the importance of expanding the scope of AI research and implementation to include nurses, not only to alleviate burnout more equitably across professions, but also to improve documentation efficiency and the overall quality of patient care.

In examining the use of CDSS to address EHR documentation burden, Laing et al. reported valuable improvements in documentation efficiency and clinical decision-making (37). However, their study did not explicitly assess the impact of CDSS tools on clinician burnout, which leaves a gap in understanding the psychological and workplace satisfaction dimensions associated with these technologies. In parallel, LLMs have also been explored as tools for generating clinical documentation. Barak-Corren et al. evaluated the use of LLMs, specifically ChatGPT, in emergency medicine settings and found substantial gains in documentation efficiency and clinician productivity (38). Nevertheless, the study also highlighted critical concerns related to the reliability and clinical validity of AI-generated content. This highlights the ongoing need for clinician oversight to ensure both patient safety and documentation accuracy (38).

Inbox management emerged as the second major subdomain contributing to clinician burnout in this study. The volume of patient portal messages has grown dramatically in recent years, a trend significantly accelerated by the COVID-19 pandemic (39, 40). This increase in asynchronous communication has placed increasing pressure on healthcare systems and providers. Consequently, inbox management burden contributes to cognitive overload and emotional exhaustion. Studies indicate that the rising volume of patient messages can undermine clinician well-being, identifying message triage and response as key stressors (9, 39, 40). In addition to the increased volume, poor EHR inbox design, inefficient workflows, and inadequate prioritization mechanisms further increase the burden, leading to issues such as notification fatigue and missed critical information (9).

To address the growing burden of inbox management, research suggests that both technological and organizational strategies may be effective (9, 44, 45). Team-based care models, which distribute messaging responsibilities across clinical staff, have shown potential in reducing clinician burnout (9, 44). In addition, improving the design and usability of EHR inbox systems, particularly by involving frontline clinicians in the redesign process, can enhance workflow efficiency and reduce cognitive overload (9, 46). Early studies indicate that combining these approaches with quality improvement methods may lead to more sustainable and clinician-friendly communication systems (9, 39, 40). However, some studies have found that traditional strategies for managing inbox burden are no longer sufficient, which highlights the need for scalable and innovative solutions to support clinical communication without compromising safety or empathy (39, 40).

This review identified AI-based approaches that focus on alleviating inbox management burden. These include the use of LLMs and NLP tools. For example, researchers at Stanford Health Care implemented a GPT-4 system to generate draft replies to patient messages, which aimed to reduce clinician workload and cognitive strain (39). Similarly, Yang et al. developed a BERT-based NLP model that automatically prioritizes high-acuity patient messages with the aim of reducing the risk of missed critical information and improving triage efficiency (40). While AI tools show promise in reducing inbox management burden, they also raise important concerns. Research points out the risk of reduced personalization in AI-generated responses, which may negatively affect patient-clinician relationships and the overall quality of care (39). Similarly, another study reported challenges regarding the completeness and clinical accuracy of AI-generated triage outputs (40). Their findings indicate the need for a hybrid approach that combines automated systems with human oversight to ensure clinical safety and data reliability (40).

Moreover, several additional concerns related to the integration of AI remain to be addressed. The use of AI in healthcare raises critical ethical and practical challenges that warrant careful consideration. A primary concern is patient data privacy, as questions persist about the extent to which AI systems can reliably safeguard sensitive health information (4, 36). This underscores the importance of data governance, particularly when AI tools are developed, owned, or managed by private entities. In such cases, it is essential to establish clear guidelines regarding who can access, use, or potentially monetize patient data (2, 9). Another pressing issue is the phenomenon of AI hallucinations, wherein systems generate inaccurate or misleading outputs (36, 38). In clinical settings, such errors could seriously compromise patient safety (36, 38). Addressing these concerns is essential to ensure that AI tools are implemented ethically, safely, and effectively within healthcare workflows.

### 4.1 Limitations of the included evidence

This systematic literature review has several limitations, both in the evidence base itself and in the review methodology.

#### 4.1.1 Limitations of the included studies

The majority of the studies included were observational in nature, such as pre-post designs, quality improvement initiatives, or pilot evaluations. These study types often lack control groups and are limited in their ability to establish causality. In many cases, outcomes were based on self-reported measures (e.g., burnout levels, satisfaction), which are subject to recall and response biases. The short follow-up periods, lack of objective metrics, and small sample sizes further reduce the robustness of the evidence. In addition, heterogeneity in AI tools, healthcare settings, and specialty areas makes cross-comparison difficult and limits generalizability.

#### 4.1.2 Limitations of the review process

This review has several limitations that may affect the comprehensiveness and generalizability of its findings. Relevant studies may have been excluded due to lack of full-text access, non-English language, or insufficient methodological detail, introducing potential language and accessibility bias. Despite a systematic search strategy, publication bias remains a concern, as studies with null or negative findings are often underrepresented. The exclusion of gray literature and preprints may have further limited the scope. A formal assessment of evidence certainty was not conducted due to the small number and heterogeneity of included studies. Moreover, meta-analysis was not feasible given the qualitative nature and variability in outcome reporting.

### 4.2 Future research

The findings of this review highlight several recommendations for future research. First, there is a need for real-world implementation studies that evaluate the long-term impacts of AI interventions across varied clinical settings. Second, multistakeholder collaboration must be prioritized in future investigations to design AI tools that are widely accepted and equitably beneficial. Third, future studies should examine the accuracy, completeness, and clinical appropriateness of AI-generated documentation and address the challenges regarding liability, privacy, and compliancy. Fourth, user group variability necessitate attention. It remains unclear which healthcare professional groups are more receptive to adopting AI documentation tools and which may face barriers due to workflow incompatibility, trust concerns, or digital literacy. Comparative studies across roles, specialties, and care settings could help tailor deployment strategies. Finally, with regard to AI use in inbox management, further research is needed to understand the balance between efficiency and personalization. While LLMs and NLP-based triage systems show promise in reducing clinician workload, concerns remain around the loss of empathy, clinical accuracy, and patient trust. Studies should evaluate how AI-generated responses affect patient satisfaction and communication quality, and test hybrid models that blend automated drafting with human oversight.

## 5 Conclusion

This systematic review demonstrates that AI technologies integrated into EHR systems hold considerable potential to alleviate burnout among healthcare professionals. Across the studies reviewed, AI interventions, particularly ambient AI scribes, CDSS, and LLM/NLP technologies, showed meaningful improvements in documentation efficiency, reduction in after-hours workload, and enhanced task management. These technologies address two primary contributors to EHR-related burnout: documentation burden and inbox management. However, while the findings suggest positive impacts on clinician workflow and, in some cases, self-reported burnout, the evidence also highlights the need for caution. Concerns regarding the accuracy, reliability, and personalization of AI-generated outputs highlights the importance of continued clinician oversight. Future research should prioritize longitudinal studies, diverse clinical settings, and robust outcome measures to assess the sustained impact and safety of AI-based interventions. Overall, AI offers a promising way to support healthcare professional well-being when thoughtfully integrated into EHR workflows.

## Data Availability

The original contributions presented in the study are included in the article/Supplementary material, further inquiries can be directed to the corresponding authors.

## References

[1] Hazarika I. Artificial intelligence: opportunities and implications for the health workforce. Int Health. (2020) 12:241–5. doi: 10.1093/INTHEALTH/IHAA007, PMID: 32300794 PMC7322190

[2] Kernberg A Gold JA Mohan V. Using ChatGPT-4 to create structured medical notes from audio recordings of physician-patient encounters: comparative study. J Med Internet Res. (2024) 26:e54419. doi: 10.2196/54419, PMID: 38648636 PMC11074889

[3] Tapuria A Porat T Kalra D Dsouza G Xiaohui S Curcin V. Impact of patient access to their electronic health record: systematic review. Inform Health Soc Care. (2021) 46:194–206. doi: 10.1080/17538157.2021.1879810, PMID: 33840342

[4] Keshta I Odeh A. Security and privacy of electronic health records: concerns and challenges. Egypt Inform J. (2021) 22:177–83. doi: 10.1016/J.EIJ.2020.07.003

[5] Wassell M Vitiello A Butler-Henderson K Verspoor K McCann P Pollard H. Electronic health Records for Predicting Outcomes to work-related musculoskeletal disorders: a scoping review. J Occup Rehabil. (2024) 34:770–82. doi: 10.1007/S10926-024-10175-1, PMID: 38536622 PMC11550283

[6] Hamad MME Bah S. Impact of implementing electronic health records on medication safety at an HIMSS stage 6 hospital: the pharmacist’s perspective. Can J Hosp Pharm. (2022) 75:267–75. doi: 10.4212/CJHP.3223, PMID: 36246440 PMC9524548

[7] Ratwani RM. Electronic health records and improved patient care: opportunities for applied psychology. Curr Dir Psychol Sci. (2017) 26:359–65. doi: 10.1177/0963721417700691, PMID: 28808359 PMC5553914

[8] Arndt BG Beasley JW Watkinson MD Temte JL Tuan WJ Sinsky CA . Tethered to the EHR: primary care physician workload assessment using EHR event log data and time-motion observations. Ann Fam Med. (2017) 15:419–26. doi: 10.1370/AFM.2121, PMID: 28893811 PMC5593724

[9] Dymek C Kim B Melton GB Payne TH Singh H Hsiao CJ. Building the evidence-base to reduce electronic health record-related clinician burden. J Am Med Inform Assoc. (2021) 28:1057–61. doi: 10.1093/JAMIA/OCAA238, PMID: 33340326 PMC8068419

[10] Hartman VC Bapat SS Weiner MG Navi BB Sholle ET Campion TR. A method to automate the discharge summary hospital course for neurology patients. J Am Med Inform Assoc. (2023) 30:1995–2003. doi: 10.1093/JAMIA/OCAD177, PMID: 37639624 PMC10654848

[11] Johnson LG Madandola OO Dos Santos FC Priola KJB Yao Y Macieira TGR . Creating perinatal nursing care plans using ChatGPT a pathway to improve nursing care plans and reduce documentation burden. J Perinat Neonatal Nurs. (2024) 39:10–19. doi: 10.1097/JPN.0000000000000831 PMC11781987 39491050

[12] Alumran A Aljuraifani SA Almousa ZA Hariri B Aldossary H Aljuwair M . The influence of electronic health record use on healthcare providers burnout. Inf Med Unlocked. (2024) 50:101588. doi: 10.1016/J.IMU.2024.101588

[13] Maslach C Leiter MP. Understanding the burnout experience: recent research and its implications for psychiatry. World Psychiatry. (2016) 15:103–11. doi: 10.1002/WPS.20311, PMID: 27265691 PMC4911781

[14] Gesner E Dykes PC Zhang L Gazarian P. Documentation burden in nursing and its role in clinician burnout syndrome. Appl Clin Inform. (2022) 13:983. doi: 10.1055/S-0042-1757157, PMID: 36261113 PMC9581587

[15] Liu H. Lou S. S. Warner B. C. Harford D. R. Kannampallil T. Lu C. (2022). HiPAL: A Deep Framework for Physician Burnout Prediction Using Activity Logs in Electronic Health Records. Proceedings of the 28th ACM SIGKDD Conference on Knowledge Discovery and Data Mining (KDD ‘22), August 14â•fi18, 2022, Washington, DC, USA, 1.

[16] Carpenter LM Swerdlow AJ Fear NT. Mortality of doctors in different specialties: findings from a cohort of 20 000 NHS hospital consultants. Occup Environ Med. (1997) 54:388–95. doi: 10.1136/OEM.54.6.388, PMID: 9245944 PMC1128798

[17] Ryan E Hore K Power J Jackson T. The relationship between physician burnout and depression, anxiety, suicidality and substance abuse: a mixed methods systematic review. Front Public Health. (2023) 11:1133484. doi: 10.3389/FPUBH.2023.1133484/FULL, PMID: 37064688 PMC10098100

[18] Budd J. Burnout related to electronic health record use in primary care. J Prim Care Community Health. (2023) 14:21501319231166920. doi: 10.1177/21501319231166921, PMID: 37073905 PMC10134123

[19] Sinsky C Colligan L Li L Prgomet M Reynolds S Goeders L . Allocation of physician time in ambulatory practice: a time and motion study in 4 specialties. Ann Intern Med. (2016) 165:753–60. doi: 10.7326/M16-0961 27595430

[20] Gardner RL Cooper E Haskell J Harris DA Poplau S Kroth PJ . Physician stress and burnout: the impact of health information technology. J Am Med Inform Assoc. (2019) 26:106–14. doi: 10.1093/JAMIA/OCY145, PMID: 30517663 PMC7647171

[21] Ahuja AS Schmidt CE. The impact of artificial intelligence in medicine on the future role of the physician. PeerJ. (2019) 7:e7702. doi: 10.7717/PEERJ.7702 31592346 PMC6779111

[22] Rashid AB Kausik MAK. AI revolutionizing industries worldwide: a comprehensive overview of its diverse applications. Hybrid Adv. (2024) 7:100277. doi: 10.1016/J.HYBADV.2024.100277

[23] Ye J Yao L Shen J Janarthanam R Luo Y. Predicting mortality in critically ill patients with diabetes using machine learning and clinical notes. BMC Med Inform Decis Mak. (2020) 20:1–7. doi: 10.1186/S12911-020-01318-4, PMID: 33380338 PMC7772896

[24] Alowais SA Alghamdi SS Alsuhebany N Alqahtani T Alshaya AI Almohareb SN . Revolutionizing healthcare: the role of artificial intelligence in clinical practice. BMC Med Educ. (2023) 23:689–15. doi: 10.1186/S12909-023-04698-Z, PMID: 37740191 PMC10517477

[25] Choudhury A Asan O. Role of artificial intelligence in patient safety outcomes: systematic literature review. JMIR Med Inform. (2020) 8:e18599. doi: 10.2196/18599, PMID: 32706688 PMC7414411

[26] Taherdoost H Ghofrani A. AI’S role in revolutionizing personalized medicine by reshaping pharmacogenomics and drug therapy. Intell Pharm. (2024) 2:643–50. doi: 10.1016/J.IPHA.2024.08.005

[27] Blackley SV Huynh J Wang L Korach Z Zhou L. Speech recognition for clinical documentation from 1990 to 2018: a systematic review. J Am Med Inform Assoc. (2019) 26:324–38. doi: 10.1093/JAMIA/OCY179, PMID: 30753666 PMC7647182

[28] Rose C Chen JH. Learning from the EHR to implement AI in healthcare. NPJ Digital Medicine. (2024) 7:330. doi: 10.1038/S41746-024-01340-0, PMID: 39567723 PMC11579417

[29] Page MJ McKenzie JE Bossuyt PM Boutron I Hoffmann TC Mulrow CD . The PRISMA 2020 statement: an updated guideline for reporting systematic reviews. BMJ. (2021) 372:1–9. doi: 10.1136/BMJ.N71, PMID: 33782057 PMC8005924

[30] Munn Z Barker TH Moola S Tufanaru C Stern C McArthur A . Methodological quality of case series studies: an introduction to the JBI critical appraisal tool. JBI Database System Rev Implement Rep. (2019) 18:2127–2133. doi: 10.11124/JBISRIR-D-19-00099 33038125

[31] Tufanaru C Munn Z Aromataris E Campbell J Hopp L. Chapter 3: Systematic reviews of effectiveness. In: Aromataris E, Munn Z, eds. JBI Manual for Evidence Synthesis. Adelaide: JBI; 2020. Available from: https://synthesismanual.jbi.global.

[32] Moola S Munn Z Tufanaru C Aromataris E Sears K Sfetcu R . (2020). Checklist for analytical cross sectional studies.

[33] Shah SJ Devon-Sand A Ma SP Jeong Y Crowell T Smith M . Ambient artificial intelligence scribes: physician burnout and perspectives on usability and documentation burden. J Am Med Inform Assoc. (2025) 32:375–80. doi: 10.1093/JAMIA/OCAE295, PMID: 39657021 PMC11756571

[34] Owens LM Wilda JJ Hahn PY Koehler T Fletcher JJ. The association between use of ambient voice technology documentation during primary care patient encounters, documentation burden, and provider burnout. Fam Pract. (2024) 41:86–91. doi: 10.1093/FAMPRA/CMAD092, PMID: 37672297

[35] Albrecht M Shanks D Shah T Hudson T Thompson J Filardi T . Enhancing clinical documentation with ambient artificial intelligence: a quality improvement survey assessing clinician perspectives on work burden, burnout, and job satisfaction. JAMIA Open. (2025) 8:ooaf013. doi: 10.1093/JAMIAOPEN/OOAF013, PMID: 39991073 PMC11843214

[36] Moryousef J Nadesan P Uy M Matti D Guo Y. Assessing the efficacy and clinical utility of artificial intelligence scribes in urology. Urology. (2025) 196:12–7. doi: 10.1016/J.UROLOGY.2024.11.061, PMID: 39622441

[37] Laing S Mercer J. Improved preventive care clinical decision-making efficiency: leveraging a point-of-care clinical decision support system. BMC Med Inform Decis Mak. (2021) 21:1–8. doi: 10.1186/S12911-021-01675-8 34763691 PMC8588582

[38] Barak-Corren Y Wolf R Rozenblum R Creedon JK Lipsett SC Lyons TW . Harnessing the Power of generative AI for clinical summaries: perspectives from emergency physicians. Ann Emerg Med. (2024) 84:128–38. doi: 10.1016/J.ANNEMERGMED.2024.01.039, PMID: 38483426

[39] Garcia P Ma SP Shah S Smith M Jeong Y Devon-Sand A . Artificial intelligence-generated draft replies to patient inbox messages. JAMA Netw Open. (2024) 7:E243201. doi: 10.1001/JAMANETWORKOPEN.2024.3201, PMID: 38506805 PMC10955355

[40] Yang J So J Zhang H Jones S Connolly DM Golding C . Development and evaluation of an artificial intelligence-based workflow for the prioritization of patient portal messages. JAMIA Open. (2024) 7:ooae078. doi: 10.1093/JAMIAOPEN/OOAE078, PMID: 39156046 PMC11328532

[41] Oxentenko AS Manohar CU McCoy CP Bighorse WK McDonald FS Kolars JC . Internal medicine residents’ computer use in the inpatient setting. J Grad Med Educ. (2012) 4:529–32. doi: 10.4300/JGME-D-12-00026.1, PMID: 24294435 PMC3546587

[42] Sutton DE Fogel JR Giard AS Gulker LA Ivory CH Rosa AM. Defining an essential clinical dataset for admission patient history to reduce nursing documentation burden. Appl Clin Inform. (2020) 11:464. doi: 10.1055/S-0040-1713634, PMID: 32643778 PMC7344356

[43] Willard-Grace R Knox M Huang B Hammer H Kivlahan C Grumbach K. Burnout and health care workforce turnover. Ann Fam Med. (2019) 17:36–41. doi: 10.1370/AFM.2338, PMID: 30670393 PMC6342603

[44] Smith CD Balatbat C Corbridge S Dopp AL Fried J Harter R . Implementing optimal team-based care to reduce clinician burnout. NAM Perspect. (2018) 8:1–13. doi: 10.31478/201809C

[45] Wright AA Katz IT. Beyond burnout — redesigning care to restore meaning and sanity for physicians. N Engl J Med. (2018) 378:309–11. doi: 10.1056/NEJMp1716845 29365301

[46] Murphy DR Satterly T Giardina TD Sittig DF Singh H. Practicing clinicians’ recommendations to reduce burden from the electronic health record inbox: a mixed-methods study. J Gen Intern Med. (2019) 34:1825–32. doi: 10.1007/S11606-019-05112-5 31292905 PMC6712240

